# Fatal Ogilvie’s syndrome after hip surgery and review of the literature

**DOI:** 10.1007/s12024-022-00470-9

**Published:** 2022-03-08

**Authors:** Diego Aguiar, Tony Fracasso, Christelle Lardi

**Affiliations:** University Center of Legal Medicine (CURML), Geneva University Hospitals and University of Geneva, Geneva, Switzerland

**Keywords:** Ogilvie’s syndrome, Acute colonic pseudo-obstruction, Autopsy, Post-mortem

## Abstract

Ogilvie’s syndrome refers to a massive dilation of the colon without mechanical obstruction. Although this syndrome is well-known in the clinical literature and may sometimes be encountered as a complication of abdominal, pelvic, or hip surgery, it has only been reported sporadically in the forensic literature. We present the case of a forensic autopsy carried out on a patient whose death was related to cecal necrosis with acute peritonitis due to Ogilvie’s syndrome following hip surgery. This diagnosis was based on clinical data, post-mortem imagery, autopsy findings, histological analysis, post-mortem chemistry, and microbiological analysis. A review of the literature and possible physiopathology of this disease are performed, while focusing on medico-legal perspectives.

## Introduction

Ogilvie’s syndrome (OS) or acute colonic pseudo-obstruction refers to distention of the colon without evidence of mechanical obstruction [1, 2]. It was first described in 1948 by Heneage Ogilvie who treated two patients presenting with symptoms suggesting distal colonic obstruction due to carcinoma. Exploratory laparotomy did reveal dilation though without any obstruction [3]. In both cases, a malignant infiltration involving the region of the coeliac axis was found, suggesting a lesion to the sympathetic extrinsic enteric nervous system. Ever since, OS has mainly been described in hospitalized patients featuring various clinical scenarios such as surgical intervention, trauma, infection, cardiac disease, neurological disease, and other miscellaneous medical conditions (metabolic, drug-induced) [4]. Clinical presentation of this syndrome has been extensively studied and includes abdominal pain and tenderness, nausea, vomiting, and constipation [4]. However, nausea and vomiting are absent in one third of cases [5]. This diagnosis can be suggested by the clinical picture and confirmed by imaging, showing dilation of the colon [6]. Differential diagnosis of OS mainly comprises mechanical obstruction of the colon (e.g., tumor, volvulus, diverticulitis, Crohn’s disease) and other types of functional obstruction such as toxic megacolon and paralytic ileus [1]. The most devastating complications of OS are cecal necrosis and perforation, which can lead to death in 46% of cases [6, 7]. Indeed, Wojtalik et al. reported that the cause of death may be either colonic perforation or cecal necrosis [7]. Most frequently, death is due to the association of both conditions (43%) or colonic perforation alone (43%) rather than cecal necrosis alone (14%).

Even though this syndrome is well-known in the clinical literature, it has seldom been reported in the forensic literature [8, 9], as well as in the clinical literature with a focus on potential medico-legal consequences [10]. Here, we present the case of a patient whose death was attributed to cecal necrosis with acute peritonitis due to Ogilvie’s syndrome following hip surgery. Given the fact that hospital deaths occurring shortly after surgery are frequently referred to forensic services to rule out any medical malpractice, this article aims to draw this post-operative complication to the medical examiners’ and forensic pathologists’ attention. We also recommend that in such cases, post-mortem imaging, along with autopsy, histology, as well as toxicological and microbiological analysis be performed.

## Case presentation

A 67-year-old man was admitted to the hospital for an elective total right hip replacement surgery in the context of osteoarthritis. This patient was known to be overweight (body mass index 29 kg/m^2^), with other comorbidities such as active smoking, dyslipidemia and arterial hypertension. Surgery was performed under general anesthesia with an anterior approach, without immediate surgical complication. The intervention lasted for an hour and fifteen minutes. Post-operative pain was managed with opioids. Additional post-operative medications included enoxaparin, ibuprofen, oxazepam and paracetamol. One day after surgery, the patient developed abdominal pain and tenderness, along with the absence of stool passage and diminished bowel sounds. Clinicians deemed those findings consistent with postoperative paralytic ileus. Treatment was then initiated, with rectal enema, laxative and fasting, though no parenteral nutrition nor gastric aspiration were introduced. Also, opioid treatment was withdrawn. On the third post-operative day, he further developed hypotension and tachycardia. Laboratory values then showed leukocytosis (18 G/l, range 4 to 11 G/l). Abdominal plain radiography revealed dilation of small intestine loops (diameter of 6 cm) with multiple air-fluid levels. On the fourth day, his clinical state worsened with desaturation and tachypnea. At that time, laboratory workup revealed increased C-reactive protein (481 mg/l, range 0 to 10 mg/l) considered unspecific due to the postoperative state and increased leukocytosis (19.5 G/l). Subsequently, the patient developed acute confusion, followed by cardio-respiratory arrest then death. In this context, the case was reported to local authorities and the public prosecutor ordered medico-legal investigations to assess the cause and manner of death.

**Fig. 1:**
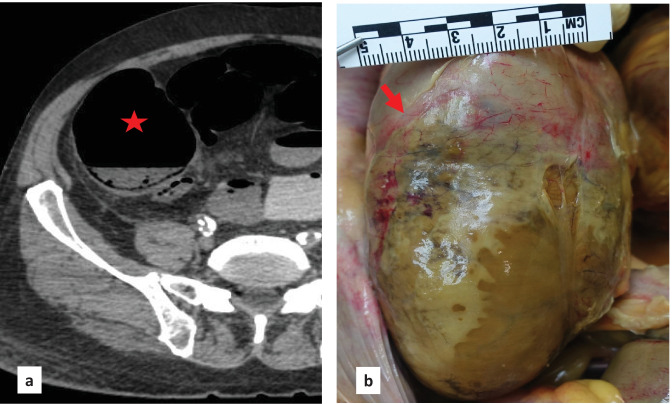
**a** Post-mortem CT-scan showing dilation of the cecum (red star). **b** Autopsy in situ view of the anterior part of the cecum presenting diffuse green-brownish discoloration with a red–purple well delineated edge (red arrow)

**Fig. 2:**
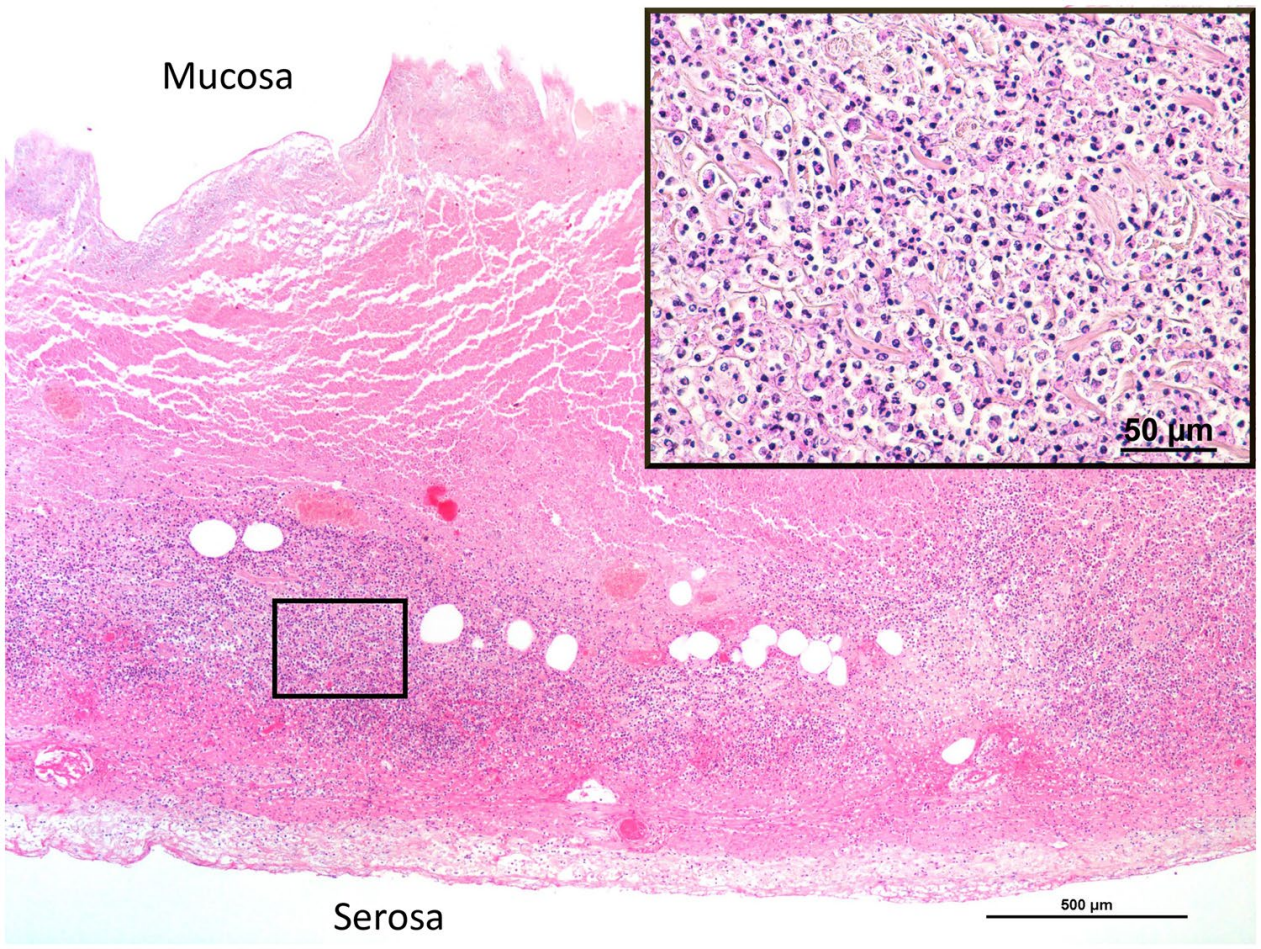
Cecal wall necrosis with diffuse polymorphonuclear neutrophil (PMN) infiltration (inset) (hematoxylin and eosin, 40 ×). Inset: Close-up view of PMN infiltration highlighted in the black box (hematoxylin and eosin, 400 ×)

**Fig. 3:**
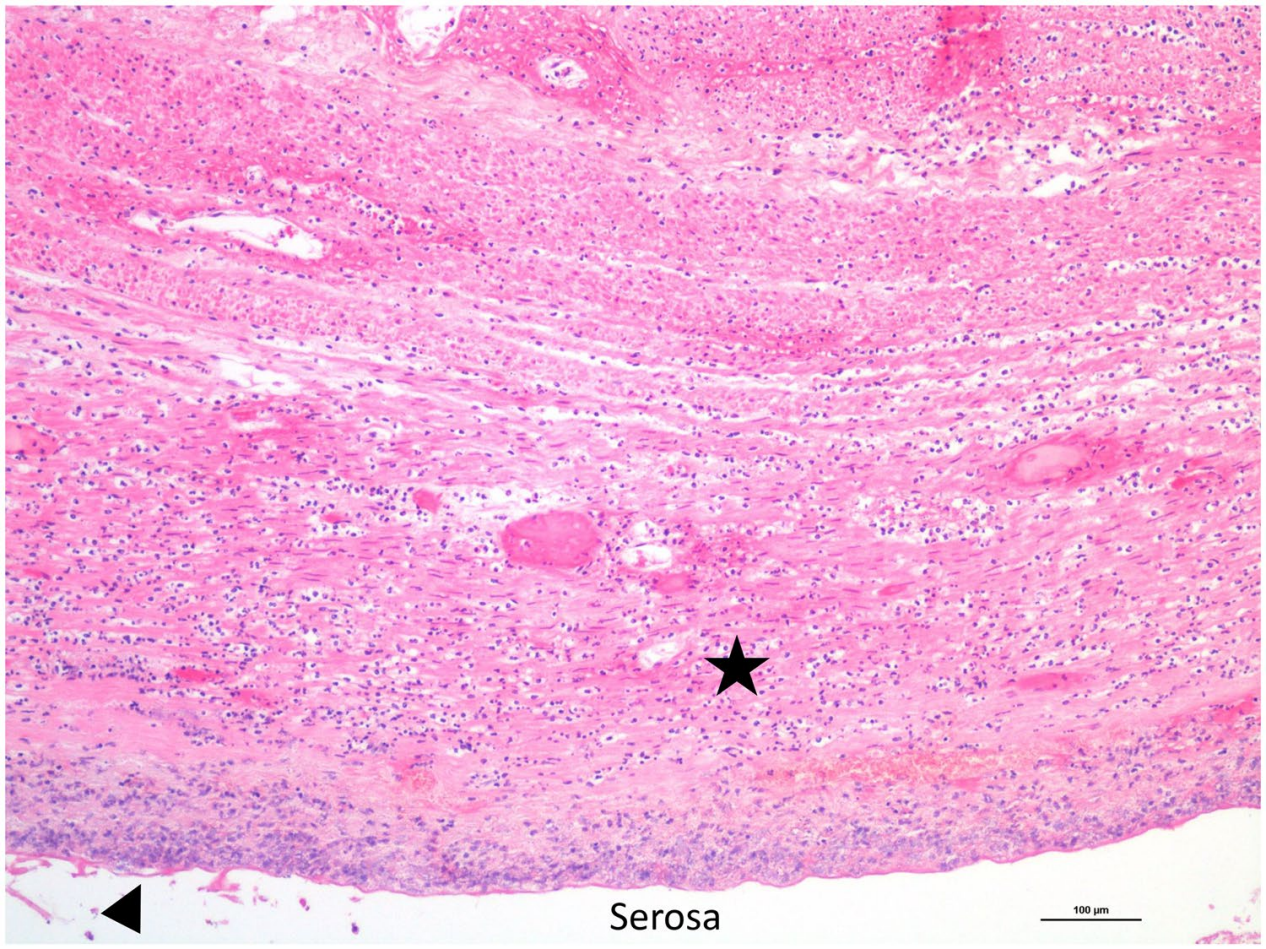
Cecal wall necrosis with diffuse polymorphonuclear neutrophil infiltration (black star) and acute peritonitis (black arrowhead) (hematoxylin and eosin, 100 ×)

Post-mortem computed tomography (PMCT) was then carried out, revealing a dilated colon up to 9 cm in diameter at the level of the cecum without pneumoperitoneum (Fig. 1a). Post-mortem radiological alteration index (RA-Index) was 13, indicating few putrefactive gases within the body [11]. A forensic autopsy was then performed on the same day, with a post-mortem interval of 43 h, confirming the presence of cecal dilation with a diameter of 9 cm. The cecal serosa was covered by yellow-brownish deposits, suggesting fibrinoid peritonitis (Fig. 1b). Those findings were also encountered on the distal segment of the terminal ileum and appendix. Under those deposits, the cecal surface showed diffuse purple and greenish discoloration and a sharp reddish demarcation with non-injured ascending colon, along with thinning of the cecal wall though without perforation. Cecal content was brownish and liquid. Furthermore, cecal mucosa exhibited the same discoloration and sharp line of demarcation as seen through the serosa. We then carried out the histological examination, showing acute inflammatory necrosis of the cecum (Figs. 2 and 3) and confirming acute fibrinoid peritonitis (Fig. 3). Submucosal plexus (Meissner’s plexus) and myenteric plexus (Auerbach’s plexus), highlighted by calretinin immunostaining, were intact in the ascending colon, distal to the cecal necrosis. Interstitial cells of Cajal (pacemaker cells of the gut wall) were also preserved in the same segments, as shown by the positive c-kit (CD117) immunostaining. Post-mortem chemistry analysis revealed an inflammatory state (C-reactive protein: 431 mg/l, range 0 to 10 mg/l) and signs of generalized bacterial infection (procalcitonin: 1.48 µg/l, range 0 to 0.25 µg/l). Post-mortem bacteriological analysis revealed the presence of mixed digestive bacterial flora within the peritoneal cavity and blood cultures. The analysis of peritoneal fluid showed various and numerous enteric bacteria such as *Escherichia coli* while blood cultures revealed a small amount of *Escherichia coli*. Moreover, examination of the operative site on the right hip revealed no gross signs of local infection and bacteriological samples remained sterile. In addition, toxicological analyses were performed on ante-mortem blood drawn 1 day after surgery and revealed the presence of oxazepam (106 µg/l) and morphine (total morphine of 153 µg/l; free morphine < 10 µg/l). The cause of death was then attributed to acute peritonitis in the context of Ogilvie’s syndrome with acute cecal necrosis. This death was considered a complication of hip surgery and no medical malpractice was retained by the public prosecutor.

## Discussion

In the field of orthopedics, Ogilvie’s syndrome has been diagnosed after elective total hip arthroplasty (THA), hip fracture, elective knee arthroplasty, and spinal surgery [5, 12–21]. In the cases of THA, surgery was complicated by OS in 0.29 to 1.6% of cases [12, 14, 16, 18, 20]. However, patients were at higher risk of developing OS when treated for trauma than for elective THA [12]. Within the subgroup of OS occurring after orthopedic surgery, the death rate reached levels of up to 13% in cases of THA [12, 14, 16]. Regarding the cause of death, ElMaraghy et al. [15] reported a fatal case following an elective right total hip arthroplasty attributed to a complication of the necrotic and perforated cecum, while Liu et al. described a fatal case after admission for left hip fracture followed by gastro-intestinal hemorrhage, heparin-induced thrombocytopenia and gut-origin septic shock [13, 15].

To this day, the physiopathology of Ogilvie’s syndrome remains uncertain and is most certainly multifactorial [22]. Numerous hypotheses were proposed, attributing the origin of OS to neural, vascular, hormonal, pharmacological, metabolic, and infectious processes [22]. Currently, the most recognized hypothesis relies on an imbalance of the autonomic nervous system regulation of the colon [22, 23]. The autonomic nervous system sympathetic nerve activation tends to inhibit gut motility while parasympathetic activation tends to activate it. Ogilvie’s original conclusion regarding the physiopathology of this syndrome was that a malignant tumor infiltrated and interrupted the sympathetic supply of the large intestine, which is then left with an uncontrolled parasympathetic drive. However, most authors now believe that it is a decrease in parasympathetic activity that creates an adynamic colon, which dilates its proximal parts [22]. In addition, few studies also suggest that hyper activation of the sympathetic system or loss of interstitial cells of Cajal could be responsible for OS [24, 25]. In the present case, it remains uncertain whether the short interval following the right total hip arthroplasty could play a role in the development of cecal necrosis. The fact that OS has been reported in THA on both the right and left sides suggests that local influence is not a decisive factor [5, 13, 15, 19].

It is worth mentioning a recent hypothesis suggesting that micro-thrombosis and hypoperfusion of the bowel wall might lead to OS. This hypothesis was based on one case of OS that occurred after discontinuation of a patient’s low molecular weight heparin treatment without switching to non-heparin anticoagulant because of severe gastrointestinal hemorrhage [13]. However, in the current case, this hypothesis can be excluded because no thrombosis was found during the autopsy and the patient was still receiving enoxaparin when OS developed.

In our case, OS developed after elective right THA due to degenerative osteoarthritis, as most frequently described in the literature, and with a comparable clinical presentation [12, 14, 17]. We hypothesize that marked dilation of the cecum generated high pressure over small arteries in the bowel wall, resulting in ischemic necrosis of the cecum. The latter led to a local spread in the peritoneal cavity along with bacterial translocation into the bloodstream. This hypothesis is supported by the abundance of enteric bacteria such as *Escherichia coli* found within the peritoneal cavity on post-mortem cultures. A case of fatal bacterial peritonitis secondary to OS has previously been described in the literature [26]. In the aforementioned case, ante-mortem bacteriological analysis of the ascitic fluid revealed bacterial colonization with *Escherichia coli* and necropsy revealed cecal necrosis without perforation. As opposed to our case, the authors were then unable to define the infectious mechanism displayed by the causative germ to achieve infection of the peritoneal cavity.

To evaluate the involvement of the enteric nervous system in the development of OS in the current case, we investigated the integrity of the Auerbach and Meissner’s plexus as well as interstitial cells of Cajal. We found that the enteric nervous system was morphologically intact in the ascending colon, just distal to the cecal dilation and necrosis. This suggests the absence of morphological disturbance of the enteric nervous system in cases of OS. However, the possibility of physiological impairment of this system remains uncertain.

Diagnosis of OS is challenging in forensic practice. Therefore, clinical data, if available, should be thoroughly reviewed in search of potential risk factors contributing to the development of OS, such as surgical intervention, trauma, infection, drug reaction as well as a cardiac, neurological and metabolic disease. Moreover, OS should especially be suspected in cases of death following orthopedic surgery. Diagnosis should then solely be based on the presence of both colonic distention and lesion, since bowel distention alone can be encountered on post-mortem imaging and during necropsy. However, caution should be carried not to overvalue this finding as evaluating colonic distention during the autopsy is hazardous. Indeed, putrefactive gas production in the post-mortem period can exacerbate distention. For this reason, one should carefully evaluate the integrity of the colonic wall in search of perforation and early signs of ischemic necrosis. Additionally, post-mortem computed tomography could help in the diagnosis of bowel perforation if pneumoperitoneum is present. Histological analysis remains the most reliable diagnostic tool in assessing ischemic changes in the colon and would also exclude another differential diagnosis such as post-mortem dilation and colitis. Post-mortem chemistry and microbiological analysis will also help in assessing bacterial peritonitis and the general inflammatory response in relation to the cause of death. Concerning the post-mortem differential diagnosis of OS, only the other types of functional obstruction should be a matter of concern.

On the one hand, toxic megacolon should be differentiated from OS when bowel dilation occurs in association with systemic toxicity, usually in the context of inflammatory bowel disease (usually ulcerative colitis) or infectious colitis (especially *Clostridium difficile*). The diagnosis of paralytic ileus, on the other hand, should be retained when both colon and small bowel show dilation, though usually predominant in the latter.

Given the constantly increasing proportion of elderly in the general population, it is estimated that hip surgery for either degenerative or post-traumatic purposes will increase in the near future [27]. Therefore, forensic pathologists will be dealing more frequently with fatal abdominal complications of surgery such as Ogilvie’s syndrome. In such cases, the possibility of medical malpractice should be ruled out. Better recognition of this entity at necropsy will also enable future studies.

## Conclusion

Ogilvie’s syndrome is a known complication following hip surgery and must be considered in cases of death occurring in a post-operative setting. As hip surgery will increase in the near future due to the aging of the population, medical examiners and forensic pathologists will be facing this diagnosis more frequently. In those suspected cases, post-mortem imaging should be performed in search of colonic distention and perforation. During the autopsy, careful examination of the digestive tract and peritoneum should be carried out, looking for signs of perforation, necrosis, and peritonitis. Histological examination, post-mortem chemistry, and bacteriological analysis allow confirmation of the diagnosis and establishment of the cause of death.

## Key points

1. Ogilvie’s syndrome (OS) or acute colonic pseudo-obstruction refers to distention of the colon without evidence of mechanical obstruction, affecting mainly hospitalized patients admitted for surgery.
2. The most devastating complications of OS are cecal necrosis and perforation, which can lead to death.
3. OS has only been reported sporadically in the medico-legal literature, and represents a challenging diagnosis to establish in the forensic practice.
4. Since hospital deaths occurring shortly after surgery are frequently referred to forensic pathologists in order to rule out medical malpractice, forensic pathologists and medical examiners should be aware of the occurrence of this postoperative complication.
5. Post-mortem diagnosis of OS should be based on post-mortem imaging, autopsy, histology, along with toxicological and microbiological analysis.
